# The Bactericidal Efficacy and the Mechanism of Action of Slightly Acidic Electrolyzed Water on *Listeria monocytogenes’* Survival

**DOI:** 10.3390/foods10112671

**Published:** 2021-11-03

**Authors:** Huiying Li, Duo Liang, Jin Huang, Chaojing Cui, Huan Rao, Dandan Zhao, Jianxiong Hao

**Affiliations:** College of Food Science and Biology, Hebei University of Science and Technology, Shijiazhuang 050018, China; 15383113670@163.com (H.L.); liangduo1221@163.com (D.L.); huangjin202110@163.com (J.H.); ccjhhxx2021@163.com (C.C.); raohuan881210@163.com (H.R.); zdd6364@126.com (D.Z.)

**Keywords:** slightly acidic electrolyzed water, *Listeria monocytogenes*, bactericidal mechanism, food safety

## Abstract

In the present work, the bactericidal efficacy and mechanism of slightly acidic electrolyzed water (SAEW) on *L. monocytogenes* were evaluated. The results showed that the strains of *L. monocytogenes* were killed completely within 30 s by SAEW whose available chlorine concentration (ACC) was higher than 12 mg/L, and it was confirmed that ACC is the main factor affecting the disinfection efficacy of SAEW. Moreover, our results demonstrated that SAEW could destroy the cell membrane of *L. monocytogenes*, which was observed by SEM and FT-IR, thus resulting in the leakage of intracellular substances including electrolyte, protein and nucleic acid, and DNA damage. On the other hand, the results found that SAEW could disrupt the intracellular ROS balance of *L. monocytogenes* by inhibiting the antioxidant enzyme activity, thus promoting the death of *L. monocytogenes.* In conclusion, the bactericidal mechanism of SAEW on *L. monocytogenes* was explained from two aspects including the damage of the cell membrane and the breaking of ROS balance.

## 1. Introduction

Foodborne pathogens have been raising increasing concerns because they are associated with the outbreak of foodborne disease and present an extensive health burden worldwide. *Listeria monocytogenes* (*L. monocytogenes*) is one of the main foodborne pathogens threatening human health in refrigerated food and is responsible for Listeriosis in children, the elderly, pregnant women and immunocompromised populations [1]. It has been reported that *L. monocytogenes* can survive and grow under a variety of extremely adverse conditions including low temperatures (0.4–45 °C), low pH values (4–9.6) and low oxygen levels, which enables it to continue to grow and reproduce in low-temperature preserved food [2,3]. Most seriously, *L. monocytogenes* enter the human digestive system first, then go across intestinal epithelial cells, enter the liver or other organs through the blood circulation, and can produce infection in the meninges and brain substance[4,5]. Scientists found that the main harmful factors of *L. monocytogenes* included *Listeria* hemolysin O, phospholipase and some virulence proteins, and so on [6,7,8,9,10,11].

In order to reduce the microbial contamination caused by foodborne pathogens, especially *L. monocytogenes*, chlorine sanitizer such as sodium hypochlorite (NaClO) and chlorine dioxide (ClO_2_) have traditionally been recognized as possessing high disinfection efficacy in the food industry. However, a low chlorine concentration could not reduce the microbial population and a high chlorine concentration may lead to the formation of trihalomethanes and haloacetic acids, which are potentially carcinogenic and teratogenic [12,13,14]. Lan et al. (2019) reported that the application of SAEW could cause a sublethal injury to the recovery of *Listeria monocytogenes*, and Jeon et al. [15] reported that SAEW can significantly reduce Listeria innocua biofilm cells on food contact surfaces during food processing. As an alternative to the traditional chlorine sanitizer, the strong antibacterial effect of electrolyzed oxidizing water (EOW) has been recognized as and has shown a promising prospect in the food industry.

EOW has two main types including strong acidic electrolyzed water (AEW) and slightly acidic electrolyzed water (SAEW) [16,17]. In the past few years, the disinfection efficacy and mechanism, as well as the application in the food industry, of AEW have been reported and reviewed in many studies [12,18,19]. Furthermore, increasing attentions have been paid to the bactericidal activities of SAEW in recent years because SAEW has a nearly neutral pH of 5.0–6.5 and lower available chlorine concentration (ACC) of 10–30 mg/L compared with AEW, thus demonstrating the advantages of SAEW in comparison with AEW [16,20,21,22]. Generally, the recent studies on SAEW have mainly focused on its application in the disinfection of food materials such as fruits, vegetables, sea foods and so on [23,24,25]. However, few studies about the mechanism of the bactericidal efficacy of SAEW have been systematically reported.

In previous studies, scientists found that the chlorine compounds in EOW are involved in hypochlorous acid (HClO), hypochlorite ion (OCl^−^) and chlorine gas (Cl_2_), which are regulated by pH, and HClO became the main form with a pH of 5.0–6.5 [16,17,18,19,20,21,22]. Thus, it was recognized that the strong disinfection efficacy of SAEW was attributed to the existence of HClO, which is 80 times more effective as a sanitizer than an equivalent concentration of the hypochlorite ion (OCl^−^) in the inactivation of bacteria [13,26]. On the other hand, researchers investigated the disinfection mechanism of SAEW on *Staphylococcus aureus* and suggested that the disinfection mechanism on *Staphylococcus aureus* cells of SAEW was disrupting the permeability of the cell membrane and the cytoplasmic ultrastructures [27]. Further research evaluated the disinfection mechanism of SAEW on *Escherichia coli* and *Staphylococcus aureus,* and the results showed that the disinfection behavior was associated with multiple cellular targets including both cell barriers and intracellular components [22]. Our previous study confirmed that SAEW could damage the cell membrane, thus causing the leakage of protein, DNA, RNA and ATP, which suggested that the differences in antibacterial efficacy between SAEW and AEW could be explained by the different impact on RNA of *Escherichia coli* and *Staphylococcus aureus* [28]. However, the bactericidal mechanism of SAEW on *L. monocytogenes* is still unknown.

The aim of the present study was to investigate the bactericidal efficacy and mechanism of SAEW on *L. monocytogenes* to help to provide results to further research on the target site for the bactericidal mechanism of SAEW. In the present study, the bactericidal effect of SAEW with different ACC on *L. monocytogenes* was firstly determined by plate count, and then the leakage of intracellular material including K^+^, intracellular protein and nucleic acid were investigated. Moreover, the DNA damage of SAEW-treated *L. monocytogenes* was evaluated by flow cytometry and the damage of the cell membrane was observed by scanning electron microscopy (SEM) and Fourier-transform infrared spectroscopy (FTIR). In addition, the levels of antioxidant enzymes including superoxide dismutase (SOD), catalase (CAT) and glutathione peroxidase (GSH-PX) in SAEW-treated *L. monocytogenes* were assayed, and the reactive oxygen species (ROS) were evaluated by laser scanning confocal microscopy (LSCM).

## 2. Materials and Methods

### 2.1. Preparation of SAEW

**Figure 1:**
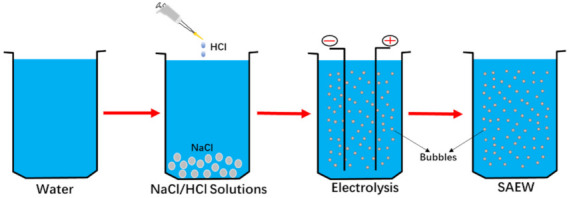
Preparation of slightly acidic electrolyzed water (SAEW) by self-made generator. SAEW was generated by passing sodium chloride and hydrochloric acid solutions through the electrolytic chamber of non-membrane.

SAEW was prepared by using a self-made electrolyzed water device and the preparation process is shown in Figure 1. After generation, SAEW was transferred to polypropylene containers and stored in dark. The pH value was determined by a pH meter (Model 86802, Orion Inc. Boston, MA, USA) and the ACC was measured using the iodometric method. The ACC of the SAEW used in this study are shown in Table 1.

### 2.2. Preparation of Bacterial Strains and Culture

*L. monocytogenes* (ATCC19114) was obtained from Solarbio (Beijing Solarbio Life Science Co., Ltd., Beijing, China) and the cultures were kept in a glass tube at −80 °C. Frozen *L. monocytogenes* was streaked in TSB-YE agar broth, then a single colony of *L. monocytogenes* was inoculated in 100 mL TSB-YE liquid broth (Beijing Land Bridge Technology, Co., Ltd., Beijing, China), and incubated at 37 °C in an orbital shaker (ZWY-100H, Shanghai Zhicheng Analytical Instruments Manufacturing Co., Ltd., Shanghai, China) at 180 r/min for 24 h.

*L. monocytogenes* suspension was centrifuged at 8000 r/min for 10 min. The sediment was collected and washed by sterile PBS twice, resuspended by sterile saline to obtain a final cell concentration approximately of 10^7^ CFU/mL. The absorbance of the bacterial suspension was measured at 450 nm using a spectrophotometer (Model Tu-1901, Beijing Purkinje General, Beijing, China) to estimate the bacterial concentration.

### 2.3. Determination of Antibacterial Effect of SAEW

An amount of 1 mL of inocula of 10^7^ CFU/mL of *L. monocytogenes* was added to 9 mL of different SAEW for different time lengths. After gradient dilution, the surviving cells were counted by spread-plating 0.1 mL of the sample or dilution onto LB (Luria-Bertani) medium, which is composed of tryptone (10g/L, Oxoid Co., Ltd., Basingstoke, Hampshire, UK), yeast extract (5 g/L, Oxoid Co., Ltd., UK), agar power (2.0% (*w*/*v*), Oxoid Co., Ltd., UK) and NaCl (10 g/L, Aoboxing Bioscience Inc., Beijing, China). The plates were incubated at 37 °C for 48 h and then the colonies were counted. In this study, the bacteria counts were expressed in log_10_CFU/mL.

### 2.4. Observation of Cell Membrane by SEM and FT-IR

*L. monocytogenes* suspension was centrifuged at 8000 r/min for 10 min and the sediment was collected and washed by sterile PBS for twice. Based on the above results of the antibacterial efficacy of SAEW on *L. monocytogenes* strains, the SAEW3 with ACC of 6.03 ± 0.13 and pH of 5.76 ± 0.03 was used in the following measurement. In brief, 1 mL of inocula of approximately 10^7^ CFU/mL of *L. monocytogenes* was added to 9 mL different SAEW3 solutions for 60 s, 0.1% sodium thiosulfate solution was used to suspend the residual activity of SAEW and then centrifuged at 8000 r/min for 10 min. The same treatment with sterile saline was used as control.

After centrifugation, the sediment was fixed with 2.5% glutaraldehyde overnight, dehydrated in an ascending acetonitrile series (50%, 70%, 80%, 90% and 100% twice for 20 min each) and dried in a lyophilizer (LGJ-10D, Four-Ring Science Instrument Plant Beijing Co., Ltd., Beijing, China). The sample power was sputter-coated with gold and observed with a scanning electron microscope (SEM, S-4800, Hitachi Co., Tokyo, Japan). The sample power was detected using a Fourier-transform infrared spectroscopy (FTIR, Nexus 670, Nicolet co., Madison, WI, America) by attenuated total reflection (ATR) with the following detection conditions: wave number range of 4000–500 cm^−1^, resolution ratio of 4 cm^−1^ and scanning numbers of 32 times.

### 2.5. Measurement of Leakage of Bacterial Intracellular Materials

The preparation of samples and bacterial suspension is described in Section 2.4. After centrifugation, the supernatant was used to take further measurements. The K^+^ and protein concentration was measured by K^+^ concentration assay kit and BCA protein assay kit (Jiancheng Bioengineering Institute, Jiangsu, China), respectively. The nucleic acid content was determined by OD_260_ value with UV spectrophotometer (UV-5200, Shanghai Yuanxi instrument Co. Ltd., Shanghai, China). The sterile saline treatment was used as control.

### 2.6. Evaluation of DNA Damage by Flow Cytometry (FCM)

The DNA damage of *L. monocytogenes* was determined using FCM after acridine orange (AO) staining. The preparation of samples and bacterial suspension was performed as described in Section 2.4. Briefly, 170 μL of 0.1 mg/mL AO staining solutions were added to SAEW-treated suspension and the mixtures were incubated at 37 °C for 10 min in dark, then tested by FCM (Accuri C6, BD Biosciences Co., San Jose, CA, USA). The sterile saline treatment was used as control.

### 2.7. ROS Analysis with Laser Scanning Confocal Microscopic (LSCM)

ROS assay kit (Beijing Solarbio Life Science Co., Ltd., Beijing, China) was used to estimate the intracellular ROS level. The preparation of samples and bacterial suspension is referred to in Section 2.4. A total of 10 μL of DCFH-DA solution was added to samples and control, respectively. The mixtures were incubated at 3 °C for 20 min with every 3-min vortex. After that, the sample was collected by centrifugation of 8000× *g* r/min for 10 min and washed with 1 mL of sterile saline solution to remove excess DCFH-DA. Finally, samples were observed with CLSM (TCS SP8, Leica Co., Wetzlar, Germany). The excitation wavelength was 488 nm and the emission wavelength was 525 nm.

### 2.8. Measurement of Intracellular Antioxidant Enzyme Activity

In the present study, three kinds of antioxidant enzymatic activity were measured including SOD, CAT and GSH-PX. The preparation of samples and bacterial suspension is referred to in Section 2.4 and the sterile saline treatment was used as control. The sediment was collected for subsequent treatment. The sediment was resuspended with saline and homogenated by a tissue homogenate machine (Pro 250, pro scientific Co., Oxford, CT, America) for 2 min. The extract was kept in −8 °C for further tests.

The activities of SOD, CAT and GSH-PX were determined by assay kits. All the assay kits were purchased from Jiancheng company (Jiancheng Bioengineering Institute, Jiangsu, China). The results were expressed as U/mg.

### 2.9. Statistical Analysis

The experiments were performed in triplicate and the data were presented as mean ± standard deviation. Data were analyzed by Duncan’s multiple range tests using SPSS software (SPSS16.0 for Windows). Significant differences between treatments were established at a significance level of *p* < 0.05. 

## 3. Results and Discussions

### 3.1. Bactericidal Efficacy of SAEW on Strains of L. monocytogenes

The bactericidal efficacy of SAEW with different ACC on *L. monocytogenes* was evaluated in the present study and the survival populations of *L. monocytogenes* are shown in Table 1. The initial counts of *L. monocytogenes* were about 7.4 log_10_ CFU/mL. From Table 1, the results showed that SAEW with ACC of more than 12 mg/L (SAEW1 andSAEW2) had significant disinfection efficacy, by which the strains of *L. monocytogenes* were completely killed within 30 s. However, the disinfection efficacy on *L. monocytogenes* of SAEW was decreased significantly when the ACC of SAEW was reduced to about 6 mg/L (SAEW3) because the time to completely kill *L. monocytogenes* was extended to 150 s. Furthermore, as the ACC deceased to less than 5 mg/L (SAEW4, SAEW5 and SAEW6), the survival counts of *L. monocytogenes* decreased by less than 1 log_10_ CFU/mL, respectively, in 150 s. Undoubtedly, HClO is the main form of the chlorine compounds in SAEW due to its pH of 5.0–6.5 compared to OCl^−^ or Cl_2_. However, the current method used to determine the chlorine compounds concentration is the iodometric method, which could not accurately determine the HClO contents. Considering that all of the types of SAEW used in the present study have similar pH values but different ACC, we confirm that ACC is the main factor affecting the disinfection efficacy of SAEW on *L. monocytogenes,* which is in agreement with the previous reports [29]

The strong bactericidal efficacy of SAEW has been recognized by many studies and SAEW has shown that it is a promising prospect in the food industry [20,29,30,31]. Our present study also demonstrated the strong bactericidal efficacy of SAEW on the strains of *L. monocytogenes.* It was reported that the bactericidal activity of SAEW against *Vibrio vulnificus* was reduced when the ACC of SAEW was less than 15 mg/L but was maintained in the *Vibrio parahaemolyticus* when the ACC of SAEW was 0.5 mg/L, which suggested SAEW with an extremely low ACC (even 0.5 mg/L) had still strong bactericidal activity [30]. The results obtained from the present study partly disagree with the previous report. In addition, it has been recognized that SAEW treatment with low ACC could not only cause the sublethal injury of *L. monocytogenes* and *E. coli* O157:H7 cells [32,33,34] but also result in the formation of viable but nonculturable (VBNC) on *E. coli* O157:H7, *Salmonella Enteritidis* and *Yersinia enterocolitica* [27,35,36], which set the new challenge to find the antibacterial activities of SAEW. Generally, the bactericidal efficacy of SAEW on *L. monocytogenes* needs further research.

### 3.2. Effect of SAEW on Intracellular Material Leakages of L. monocytogenes

**Figure 2:**
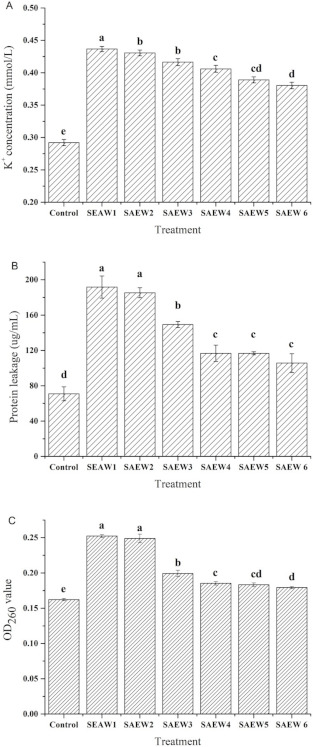
Effect of SAEW(slightly acidic electrolyzed water) on the leakage of intracellular material in *L. monocytogenes* including (**A**) K^+^ concentration, (**B**) intracellular protein and (**C**) intracellular nucleic acid. SAEW with different ACC(available chlorine concentration.) were used for 60 s in the experiment and non-treated *L. monocytogenes* as control. Each experiment was performed in triplicate and data were presented as mean ± SD (standard deviation). Different letters on the top of the bar mean significant difference between different samples (*p* < 0.05).

The cell membrane is an important structure of bacteria, which has the function of controlling material exchange. The intracellular material would leak when the cell membrane was damaged, which would lead to bacterial death [37,38]. As shown in Figure 2, SAEW treatment resulted in the remarkable increase in the extracellular levels of K^+^ concentration (Figure 2a), proteins (Figure 2b) and nucleic acids (Figure 2c), which indicated that the cell membrane of *L. monocytogenes* had been injured due to the leakage of intracellular K^+^, protein and nucleic acids.

It can be seen from Figure 2 that the concentration of each intracellular material in the control was the lowest. After SAEW treatment, the leakage concentration of each intracellular material was increased, and the leakage was positively correlated with the concentration of ACC in the SAEW. Moreover, as the results of the bactericidal effect showed, it was found that the leakage of intracellular material corresponded to the death of *L. monocytogenes*. It indicated that the cell membrane of dead *L. monocytogenes* was damaged and SAEW destroyed the cell membrane of *L. monocytogenes,* which is similar to the previous studies [27,28].

### 3.3. Effect of SAEW on Cell Membrane of L. monocytogenes

**Figure 3:**
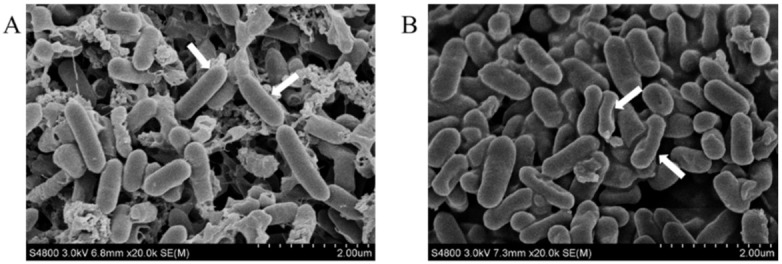
Morphological changes of *L. monocytogenes* treated by SAEW(slightly acidic electrolyzed water): (**A**) Control SEM image and (**B**) SAEW treatment SEM image. SAEW with ACC(available chlorine concentration) of 6.03 ± 0.13 and pH of 5.76 ± 0.03 was used for 60 s in the experiment.

Morphological changes of SAEW-treated and non-treated *L. monocytogenes* were observed using SEM. As shown in Figure 3, the body cells of *L. monocytogenes* had a complete morphology, smooth surface and regular texture in the control. However, the images of *L. monocytogenes* treated with SAEW revealed that the cells were markedly shrunken and partially collapsed. This phenomenon may be caused by the release of intracellular material, which was brought out by the cell membrane damage and the osmotic pressure out of balance [27]. Our previous study also found that SAEW could damage the structure and inner substances of *Escherichia coli* and *Staphylococcus aureus* [28].

**Figure 4:**
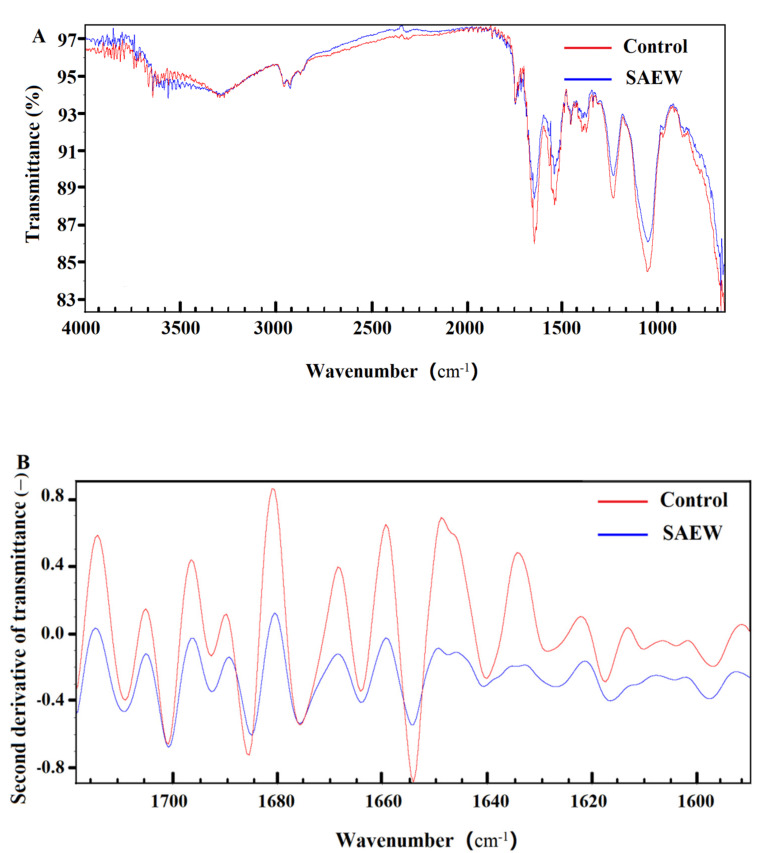
Fourier-transform infrared spectroscopy (FTIR) evaluation on the *L. monocytogenes* treated by SAEW(slightly acidic electrolyzed water) including (**A**) original FTIR graph and (**B**) the corresponding second derivative infrared spectra graph. SAEW with ACC(available chlorine concentration) of 6.03 ± 0.13 and pH of 5.76 ± 0.03 was used for 60 s in the experiment.

Moreover, the FTIR analysis was performed to verify the damage on the cell membrane of *L. monocytogenes* by SAEW, and the results are shown in Figure 4, including the original FTIR graph and the corresponding second derivative infrared spectra graph. In general, the similar peak shapes were observed between the SAEW treatment and the control, but the peak climax showed obvious difference. The results indicated that the cell membrane of *L. monocytogenes* was affected by SAEW treatment. In the present study, we focused on the range of 1680–1610 cm^−1^, which is assigned to the bond of C=C [39]. The higher the peak is, the more content of C=C it possesses. As shown in Figure 4, the peak height of *L. monocytogenes* treated by SAEW including 1638, 1648 and 1668 cm^−1^ decreased significantly in comparison with that of the control. The results suggested that SAEW could cause the decrease in C=C and the destruction of cell physiological components, thus resulting in the damage to the cell membrane of *L. monocytogenes.*

### 3.4. Effect of SAEW on DNA Damage of L. monocytogenes

**Figure 5:**
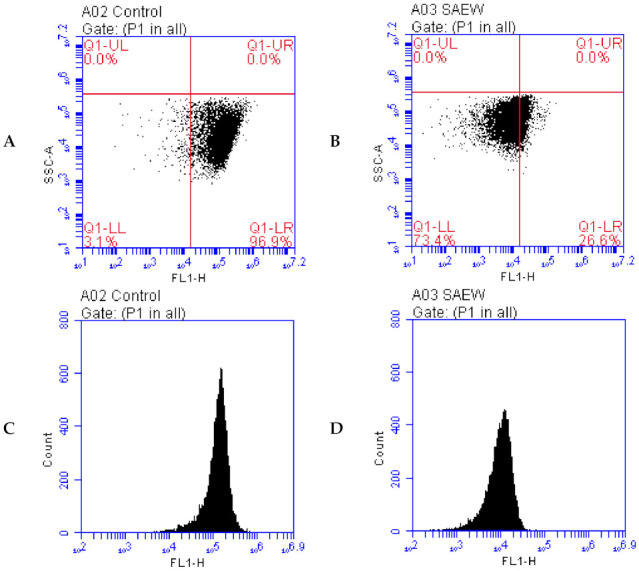
Effect of SAEW(slightly acidic electrolyzed water) on DNA damage in *L. monocytogenes* observed by flow cytometry (FCM) including: (**A**,**C**) fluorescence dot plots and counts of *L. monocytogenes* as control, (**B**,**D**) fluorescence dot plots and counts of *L. monocytogenes* treated by SAEW. SAEW with ACC(available chlorine concentration) of 6.03 ± 0.13 and pH of 5.76 ± 0.03 was used for 60 s in the experiment.

Acridine orange (AO) is a fluorochrome, which can enter the cells and combine with DNA to emit green fluorescence [40]. The fluorescence intensity can be measured by FCM to reflect the changes in DNA. The effect of SAEW on the DNA damage of *L. monocytogenes* was evaluated by FCM, and the results are shown in Figure 5. As presented in Figure 5a,b, the lower right region represents the DNA region, and the lower left region represents the damaged DNA region. It is clear that the ratio of integrated DNA in SAEW-treated *L. monocytogenes* is about 26.6% instead of about 96.9% in the non-treated strain as a control, which suggests that SAEW treatment could reduce the ratio of the integrated DNA by approximately 70%, thus resulting in the DNA damage of *L. monocytogenes.*

In Figure 5c,d, the *X*-axis represents the green fluorescence intensity, and the *Y*-axis represents the number of cells. After SAEW treatment, the fluorescence intensity of most cells was significantly reduced, and the fluorescence intensity of most cells moved from 10^5^ to 10^3^–10^4^. These results suggest that the DNA of *L. monocytogenes* was damaged by SAEW treatment. As mentioned above in the present study, SAEW caused oxidative damage in *L. monocytogenes*.

### 3.5. Effect of SAEW on Antioxidant Enzyme Activity and ROS of L. monocytogenes

**Figure 6:**
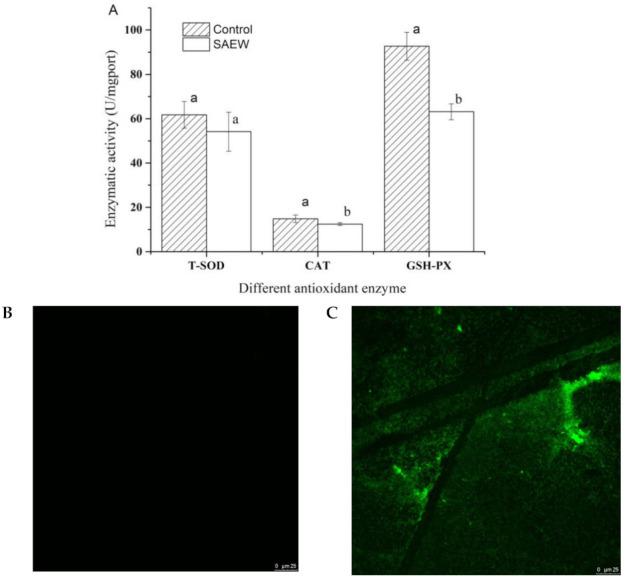
Effect of SAEW on antioxidant enzyme activity and ROS of *L. monocytogenes*. (**A**) Changes in the activity of three antioxidant enzymes including SOD, CAT and GSH-PX. Different letters on the top of the bar mean significant difference between different samples (*p* < 0.05), (**B**) LSCM image of control, (**C**) LSCM image of SAEW treatment. SAEW with ACC of 6.03 ± 0.13 and pH of 5.76 ± 0.03 was used for 60 s in the experiment.( SAEW, slightly acidic electrolyzed water; ACC, available chlorine concentration; CAT, catalase; GSH-PX, glutathione peroxidase; ROS, reactive oxygen species; LSCM, scanning confocal microscopy).

SOD and CAT are the two key antioxidant enzymes that remove ROS. SOD catalyzed O^2−^ into H_2_O_2_ by the reaction of 4O^2−^ + 2H^+^ = H_2_O_2_ + O_2_ and H_2_O_2_ is further decomposed into H_2_O by CAT. GSH-PX is an important peroxidase widely found in the organism, which could eliminate hydroperoxides and reduce the toxicity of peroxides to non-toxic hydroxyl compounds. Generally, SOD, CAT and GSH-PX contribute to the enzyme antioxidant defense system together, which were able to effectively remove reactive oxygen radicals [41,42]. Therefore, the activity of SOD, CAT and GSH-PX in the cells of *L. monocytogenes* was measured in the present work and the results are shown in Figure 6a. There were no significant changes in the SOD activity of the cells of *L. monocytogenes* between the SAEW treatment and control (*p* > 0.05). However, the activities of CAT and GSH-PX in the cells of *L. monocytogenes* treated by SAEW were decreased significantly compared with those of the control (*p* < 0.05). These results suggest that the first step of the antioxidant chain may have no obvious change and the ROS will be converted to H_2_O_2_ by intracellular SOD in the cells of *L. monocytogenes* treated by SAEW, but the CAT and GSH-PX activity under the influence of SAEW dropped significantly and, thus, inhibited the further decomposition from H_2_O_2_ into H_2_O and O_2_.

It was reported that SAEW did not result in the accumulation of reactive oxygen species (ROS) inside the microbial cell, indicating that SAEW conducted a ROS-independent behavior on the microbial inactivation, and the chemical oxidants (e.g., hypochlorous acid) played a major role in the microbial intracellular oxidation processing [27]. However, generally, our results show that SAEW treatment could destroy the balance of ROS, which is inconsistent with the previous study. We deduced that the disinfection mechanism of SAEW may be different due to the differences among the bacterial species and this needs further research.

The bacteria could be stained with DCHF-DA, which could combine with ROS to produce green fluorescence. The accumulations of ROS in the cells of *L. monocytogenes* were evaluated by the laser scanning confocal microscopic (LSCM). As shown in Figure 6, there was almost no green fluorescence in the control (Figure 6b), which indicated that intracellular ROS in *L. monocytogenes* was at certain levels. Nevertheless, there was a large amount of green fluorescence observed in the SAEW-treated cells (Figure 6c), indicating that SAEW treatment caused a mass of intracellular ROS explosion in *L. monocytogenes*. Our present study suggests that SAEW could destroy the balance of ROS in *L. monocytogenes*, which is in agreement with the above study.

ROS were produced by the metabolism of bacterial cells. Excessive ROS would damage the function of bacteria, including affecting the stability of the DNA and inducing oxidative denaturation of lipid. Bacterial cells could manage the ROS level by a free radical scavenging system. To sum up, SAEW can break the intracellular ROS balance of *L. monocytogenes* by inhibiting the antioxidant enzyme activity. In addition, *L. monocytogenes* was able to provide a large amount of hydroxyl free radicals [27]. Thus, we deduced that SAEW could break the balance of ROS to promote the death of *L. monocytogenes* [43,44]. However, the antimicrobial signaling pathways of SAEW on *L. monocytogenes* need to be further explored.

## 4. Conclusions

In the present work, the bactericidal efficacy and mechanism of SAEW to *L. monocytogenes* has been evaluated. The results showed that SAEW had a good bactericidal effect on strains of *L. monocytogenes*, and ACC played an important role in the bactericidal efficacy. Moreover, our results demonstrated that SAEW could destroy the cell membrane of *L. monocytogenes,* thus resulting in the leakage of intracellular substances including electrolyte, protein and nucleic acid and DNA damage. On the other hand, the results found that SAEW could disrupt the intracellular ROS balance of *L. monocytogenes* by inhibiting the antioxidant enzyme activity, thus promoting the death of *L. monocytogenes.* In conclusion, the bactericidal mechanism of SAEW on *L. monocytogenes* was explained from two aspects including the damage of the cell membrane and the breaking of ROS balance. The antimicrobial signaling pathways of SAEW on *L. monocytogenes* need to be further explored.

## Figures and Tables

**Table 1:** The survival populations of *L. monocytogenes* (log_10_ CFU/mL) treated by different SAEWs for different time.

SAEW Solutions	pH Value	ACC (mg/L)	Treatment Time (s)					
			0	30	60	90	120	150
SAEW ^1^	5.73 ± 0.05 ^A^	24.81 ± 0.18 ^A^	7.34 ± 0.04 ^Aa^	0 ^Ab^	0 ^Ab^	0 ^Ab^	0 ^Ab^	0 ^Ab^
SAEW ^2^	5.75 ± 0.04 ^A^	12.35 ± 0.22 ^B^	7.28 ± 0.07 ^Aa^	0 ^Ab^	0 ^Ab^	0 ^Ab^	0 ^Ab^	0 ^Ab^
SAEW ^3^	5.76 ± 0.03 ^A^	6.03 ± 0.13 ^C^	7.45 ± 0.13 ^Aa^	5.34 ± 0.04 ^Bbc^	5.50 ± 0.20 ^Bb^	5.12 ± 0.05 ^Bc^	4.09 ± 0.09 ^Bd^	0 ^Ae^
SAEW ^4^	5.73 ± 0.05 ^A^	4.25 ± 0.38 ^D^	7.34 ± 0.04 ^Aa^	6.88 ± 0.03 ^Cb^	6.95 ± 0.07 ^Cb^	6.78 ± 0.10 ^Cbc^	6.73 ± 0.06 ^Cbc^	6.60 ± 0.15 ^Cc^
SAEW ^5^	5.80 ± 0.06 ^A^	3.54 ± 0.24 ^E^	7.37 ± 0.02 ^Aa^	7.02 ± 0.05 ^Db^	6.94 ± 0.03 ^Cbc^	6.85 ± 0.04 ^Cbc^	6.81 ± 0.10 ^Cc^	6.57 ± 0.11 ^Cd^
SAEW ^6^	5.81 ± 0.05 ^A^	2.39 ± 0.16 ^F^	7.39 ± 0.02 ^Aa^	7.11 ± 0.03 ^Eb^	6.96 ± 0.06 ^Cbc^	6.80 ± 0.10 ^Cc^	6.82 ± 0.05 ^Cc^	6.62 ± 0.10 ^Cd^

SAEW is the abbreviation of slightly acidic electrolyzed water and ACC is the abbreviation of available chlorine concentration. SAEW 1–6 means different SAEW treatments. The pH and ACC values came from treatment solutions in triplicate and the result was expressed as mean ± SD (standard deviation). Different capital letters mean significant differences in each column (*p* < 0.05). Different lowercase letters mean significant differences in each row (*p* < 0.05).

## Abbreviations

SAEW, slightly acidic electrolyzed water; ACC, available chlorine concentration; AEW, acidic electrolyzed; NaClO, sodium hypochlorite; ClO_2_, chlorine dioxide; EOW, electrolyzed oxidizing water; HClO, hypochlorous acid; OCl-, hypochlorite ion; Cl2, chlorine gas; SOD, antioxidant enzymes including superoxide dismutase; CAT, catalase; GSH-PX, glutathione peroxidase; ROS, reactive oxygen species; SEM, scanning electron microscopy; FTIR, Fourier-transform infrared spectroscopy; LSCM, scanning confocal microscopy.
